# A review of the genus *Brachytrycherus* Arrow (Coleoptera, Endomychidae) of mainland China with descriptions of three new species

**DOI:** 10.3897/zookeys.880.34712

**Published:** 2019-10-14

**Authors:** Ling-Xiao Chang, Wen-Xuan Bi, Guo-Dong Ren

**Affiliations:** 1 Science Research Department, Beijing Museum of Natural History, Beijing 100050, China Beijing Museum of Natural History Beijing China; 2 Room 401, No. 2, Lane 155, Lianhua South Road, Shanghai, 201100, China Unaffiliated Shanghai China; 3 College of Life Sciences, Hebei University, Baoding 071002, China Hebei University Beijing China

**Keywords:** Coleoptera, Endomychidae, new species, taxonomy, China

## Abstract

This paper presents a review of the genus *Brachytrycherus* Arrow from mainland China. Three new species are described and illustrated: *B. bipunctatus* Chang & Bi, **sp. nov.**, *B. denticulatus* Chang & Bi, **sp. nov.**, and *B. humeralis* Chang & Bi, **sp. nov.** The diagnosis, distribution, type locality, biology, and ecology are provided for each species. A key to the species of *Brachytrycherus* known in China is updated.

## Introduction

Arrow (1920) established *Brachytrycherus* for two new species from India, *B. perotteti* and *B. madurensis*. This genus belongs to the largest endomychid subfamily Lycoperdininae. In 2005, *Brachytrycherus* was placed in the Amphisternus-group with seven other genera by Tomaszewska (2000, 2005), based on the following characters: mesoventrite with intercoxal process widened laterally towards apex, overlapping parts of coxae, elytra with basal margins thickened and raised, mandible with apical tooth widely chisel-shaped, and male genital segment with an additional internal V- or U-shaped sclerite.

The most recent synopsis of the tribe Amphisternini (Amphisternus-group) was completed by Strohecker (1964), wherein he treated and listed four species of *Brachytrycherus*, and also provided a key to the species of *Brachytrycherus* known at that time. Strohecker (1971) then transferred two species from *Engonius* Gerstaecker to the genus *Brachytrycherus*: *E. opimus* Gorham, 1896 (= *Engonius gemmatus* Arrow, 1928) and *E. femoralis* Arrow, 1928). Shockley et al. (2009a) listed six species in the updated checklist of the entire family. Since then, Chang et al. (2016) described two new species from China, *B. conaensis* and *B. curviantennae*. Furthermore, *B. conaensis* is the first species of the Handsome fungus beetles recorded feeding on Ascomycetes. Prior to the present study, this genus included eight species (Table 1).

During the examination of the Endomychidae collected in China, three new species were recognized and are described here. The recent key of Chang et al. (2016) to species of *Brachytrycherus* known in China is modified and updated.

**Table 1.** Known species of *Brachytrycherus* and distribution.

Species	Distribution
*B. conaensis* Chang et al., 2016	China (Xizang)
*B. concolor* Arrow, 1937	Borneo
*B. convexus* Strohecker, 1964	India
*B. curviantennae* Chang et al., 2016	China (Xizang)
*B. femoralis* (Arrow, 1928)	China (Guangxi), Laos, Vietnam
*B. gemmatus* (Arrow, 1928)	Laos, Myanmar, Thailand
*B. madurensis* Arrow, 1920	China (Taiwan), India
*B. perotteti* Arrow, 1920	India

## Materials and methods

Type specimens of the new species described here are deposited in the following institutions or private collections:

**BJMNH** Beijing Museum of Natural History, Beijing, China

**CBWX** Collection of Wen-Xuan Bi, Shanghai, China

**CCLX** Collection of Ling-Xiao Chang, Beijing, China

**CSHT** Collection of Hai-Tian Song, Fujian, China

**IZCAS** Institute of Zoological, Chinese Academy of Sciences, Beijing, China

**MHBU** Museum of Heibei University, Baoding, China

**MZPW** Museum and Institute of Zoology, Polish Academy of Sciences, Warszawa, Poland

**SHNU** Shanghai Normal University, Shanghai, China

Specimens were examined and described using a Nikon SMZ800 dissecting microscope. The following measurements were made using a Leica M205A dissecting microscope: body length from apical margin of clypeus to apex of elytra; width across both elytra (at widest part); elytral length along suture, including scutellum. The aedeagus was boiled in 10% NaOH solution, cleaned, and finally dissected in distilled water. Habitus photos were taken using a Canon Eos 5D III SLR camera and Canon MP-E 65 mm macro lens. All photographs were modified in Adobe Photoshop CC 2015.

## Taxonomy

### Brachytrycherus

Taxon classificationAnimaliaColeopteraEndomychidae

Arrow, 1920

9EE5CD75-E5F7-59E8-997B-3EF86F64D5B7

Brachytrycherus Arrow, 1920: 12.

#### Type species.

*Brachytrycherus perotteti* Arrow, 1920.

#### Diagnosis.

As stated in Chang et al. (2016), species of *Brachytrycherus* resemble those of *Ohtaius* Chûjô and *Gerstaeckerus* Tomaszewska in having the body black or blackish brown, elytral maculae transverse, most often orange or yellow. These genera share the feature of having the mandibles chisel-shaped apically. However, *Brachytrycherus* can be distinguished from these genera by the following combination of characters: 1) body less elongate; 2) head with well-developed gular sutures; 3) mesoventral process with sides parallel; 4) maxillary lacinia with tuft of S-like setae apically (Tomaszewska 2005).

#### Description

(based on Tomaszewska 2005). Body squat-oval to oval, moderately convex to strongly convex, glabrous or minutely pubescent. Colour dark brown to black, usually with orange or yellow markings on elytra.

Head with gular sutures well developed, widely separated, convergent apically. Antennae (Fig. 20A–C) 11-segmented, long and slender or rather stout; antennal club 3-segmented, loose. Mandible with chisel-shaped apical tooth and moderately large subapical tooth. Maxilla with terminal palpomere longer than wide, tapering apically; lacinia with tuft of S-shaped apical spines.

Pronotum transverse, widest near 1/2 of pronotal length or apical 1/3; anterior edge with rater large stridulatory membrane; sides weakly undulate or strongly curved. Prosternal process (Fig. 21A–C) not extending beyond coxae; narrowly separates procoxae, sides weakly curved outwardly or nearly straight, rounded, weakly truncate or emarginate apically. Mesoventral process (Fig. 21A–C) transverse, lateral margins widening apically and overlapping parts of coxae. Elytra anterior edge thickened and raised; sides curved, widest near 1/2 length of elytron; most often with contrasting markings. Tibiae (Fig. 22A–C) most often with sexual characters, in male with different degrees of concavity, curved or tooth.

Abdomen in both sexes with five ventrites. Ventrite V (Fig. 23A–C) almost always with sexual characters, posterior margin in male weakly curved or rounded medially, and/or with longitudinal short wrinkles laterally. Male genital segment with paired apophyses fused along nearly 1/3 of its length basally; dorsal plate undivided; additional, internal, V-shaped sclerite present.

Aedeagus (Fig. 24A–C) rather long, heavily sclerotized, without basal curvature. Median lobe branched apically. Tegmen placed basally, ring-shaped, fused with parameres.

#### Distribution.

Oriental Region (India, Laos, Thailand, South of China).

### Brachytrycherus bipunctatus

Taxon classificationAnimaliaColeopteraEndomychidae

Chang & Bi sp. nov.

478B81D3-5548-583F-8A7D-292D588405D3

http://zoobank.org/178DF400-CB0E-4775-A034-D1B9C195226C

Figs 1 , 2 , 3 , 4

#### Type material.

**Figure 1.**
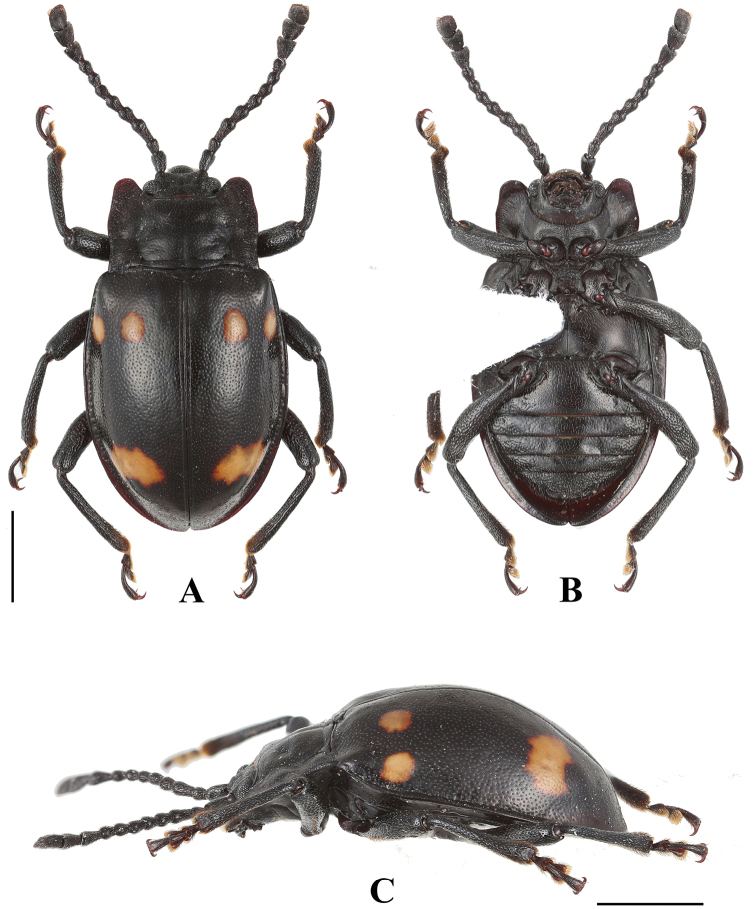
Habitus of *B. bipunctatus* sp. nov. (male). **A** dorsal view **B** ventral view **C** lateral view. Scale bar: 2 mm.

**Figure 2.**
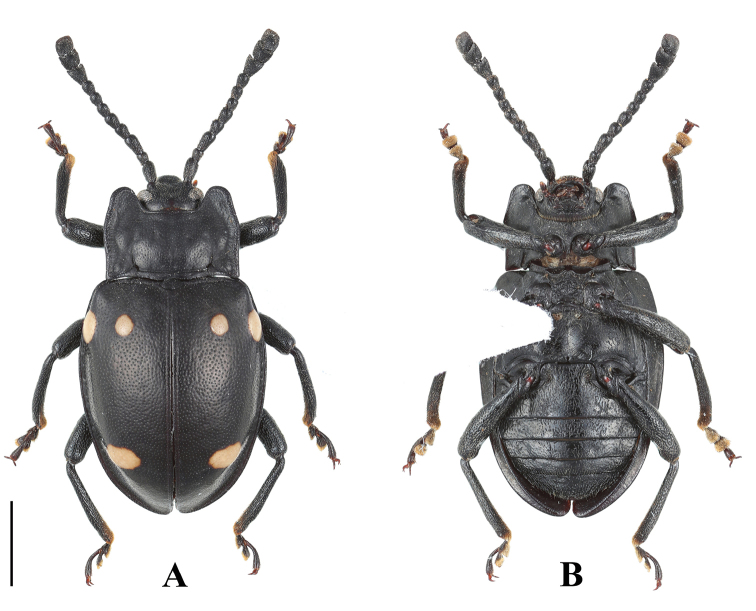
Habitus of *B. bipunctatus* sp. nov. (female). **A** dorsal view **B** ventral view. Scale bar: 2 mm.

Holotype (Fig. 1), male, **Hainan**, Fanjia Forest Reserve, 196 m, 19°16.806'N, 109°40.494'E, 17. IV.2016, Xing-Long Bai leg. (MHBU). Paratypes (Fig. 2), 1 female, Hainan, Qiongzhong, Shifangzhen, 22.II.2019, Guo-Dong Ren leg. (BJMNH); 1 female, Hainan, Dongfang, Mihouling, 11.VI.2008, Yi-Bin Ba & Jun-Tong Lang leg. (MHBU); 2 females, Hainan, Yinggeling. Nankai Township, Fangyuan Village, 21.VII.2013, Bo Cai leg. (CCLX); 1 female, **Yunnan**, Xishuangbanna, Mengla County, Mengla Town, Bubang Village, H: 680 m, 21°36'6"N, 101°35'9"E, Jian-Yue Qiu & Hao Xu leg. (CCLX) (Fig. 2).

#### Etymology.

The specific name is derived from the two apical elytral maculae.

#### Diagnosis.

*Brachytrycherus bipunctatus* can be distinctly separated from all congeners by having two distant spots on the elytral base.

#### Description.

Length 7.6–8.2 mm, width 4.1–4.2 mm. *Body* oval, approximately 2.0 times as long as wide; moderately convex; shiny. Colour black with three yellow maculae on each elytron.

**Figure 3.**
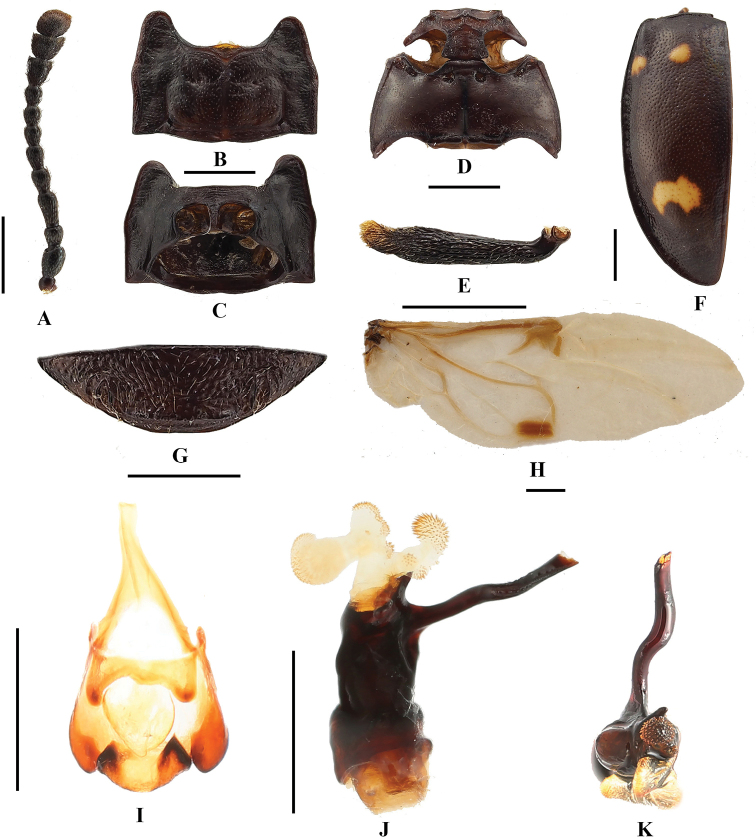
*B. bipunctatus* sp. nov. **A** antenna **B** pronotum **C** proventrite **D** male meso- and metaventrites **E** male protibia **F** elytron **G** male ventrite V of abdomen **H** hind wing **I** male genital segment **J** aedeagus in lateral view **K** aedeagus in apical view. Scale bars: 1 mm.

***Head.*** Antenna (Fig. 3A) rather stout, nearly 1/2 body length, with antennomeres 1–8 longer than wide; scape approximately 4.0 times as long as pedicel; antennomere 3 as long as 4 and 5 combined; antennomeres 4–6 nearly length equal; antennomere 6 longer than 7 and antennomere 7 as long as 8; club composed of three antennomeres, moderately broad, weakly flat, loose. Maxilla with terminal palpomere elongated, almost 1.5 times as long as palpomere 3, slightly tapering anteriorly, truncate apically.

***Thorax.*** Pronotum (Fig. 3B) 1.5–1.7 mm long, 2.7–3.1 mm wide; widest near 1/2 of pronotal length; coarsely and densely punctate; anterior and lateral margins moderately narrowly bordered; anterior edge with rather large stridulatory membrane; sides weakly undulate; front angles produced anteriorly, blunt round; disc weakly convex, two round raised area laterally; transverse wrinkle laterally; median furrow shallow, two small round pits laterally; lateral sulci linear, groove, deep, extending to basal 1/3 length of pronotum; basal sulcus nearly straight, deep. Prosternal process (Fig. 3C) not extending beyond coxae; very narrowly separates procoxae, sides weakly curved outwardly, rounded apically in male; in female rather narrowly, sides nearly straight, weakly truncate apically. Mesoventral process (Fig. 3D) transverse, lateral margins distinctly widening apically in male; in female lateral margins nearly parallel; posterior margin nearly straight.

***Elytra*** (Fig. 3F) 5.9–6.2 mm long, 3.6–3.9 times as long as and 1.4–1.5 times as wide as pronotum; sides curved, widest near 1/2 length of elytron; densely and coarsely punctate; humeri rather prominent. Each elytron with three orange-yellow maculae. Anterior two elytral maculae nearly oval or round, located behind humeri, size subequal, transverse arrangement, spacing between them subequal diameter of one macula. Posterior macula cloud-form, transverse, outer sides far from elytral lateral margin, inner margin of macula far from elytral suture. Protibia (Fig. 3E) in male with concavity on inner edge of apical 1/4 distinctly, in female without concavity; meso- and metatibiae simple. Hind wing (Fig. 3H) normal.

***Ventrite* V** (Fig. 3G) with lateral margins abruptly converging posteriorly, three or four pairs of longitudinal short wrinkles laterally; posterior margin weakly curved medially in male; in female ventrite V lateral margins gently converging posteriorly, without longitudinal wrinkles; posterior margin bluntly round medially. Male genital segment (Fig. 3I) with paired apophyses fused along nearly half of its length apically; dorsal plate undivided; additional, internal, V-shaped sclerite present.

***Aedeagus*** (Fig. 3J–K) rather long, heavily sclerotized, straight. Median lobe branched apically; branch long, in apical view longitudinal Z-shaped; truncate apically. Tegmen placed basally, comparatively large, ring-shaped, parameres rectangle, fused with tegmen.

#### Biology and ecology.

**Figure 4.**
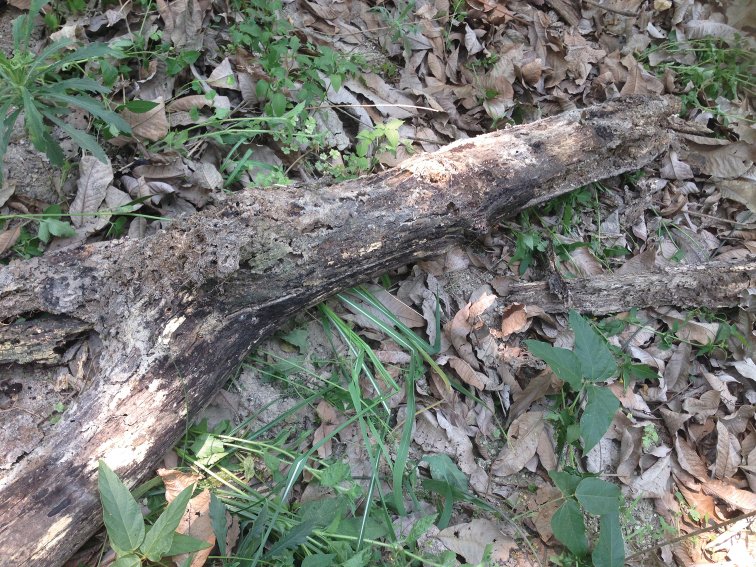
Habitats of *B. bipunctatus* sp. nov.

The holotype was hand collected by simple searching, as it is active under the fallen decayed wood in the day (Fig. 4).

### Brachytrycherus denticulatus

Taxon classificationAnimaliaColeopteraEndomychidae

Chang & Bi sp. nov.

57E68B58-1581-597E-90A0-8C7E532BB201

http://zoobank.org/E1E38FED-0977-4275-B16A-AF99873C1E60

**Figure 5.**
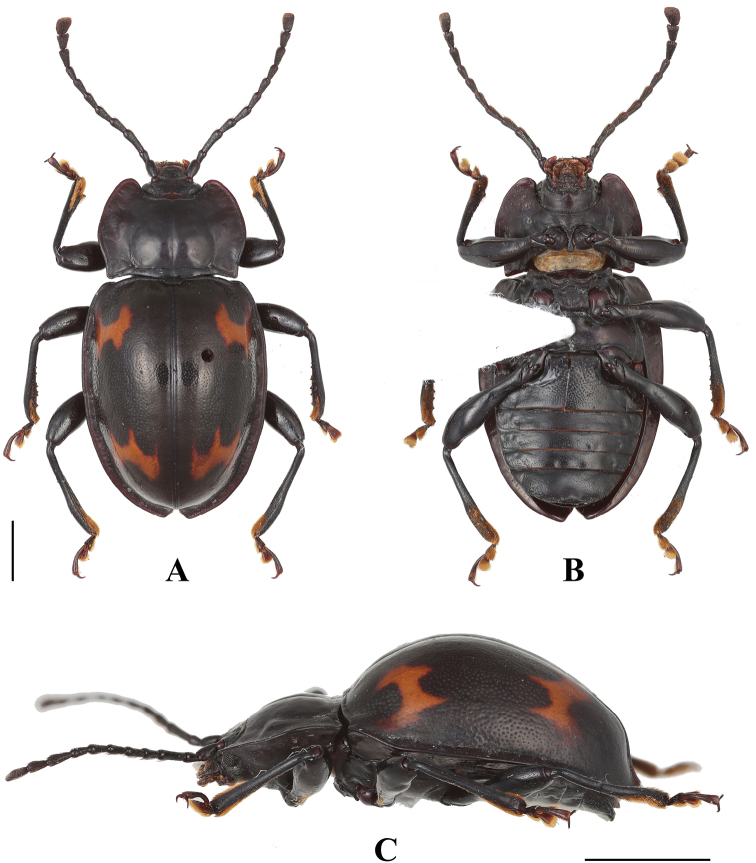
Habitus of *B. denticulatus* sp. nov. (male). **A** dorsal view **B** ventral view **C** lateral view. Scale bars: 2 mm.

**Figure 6.**
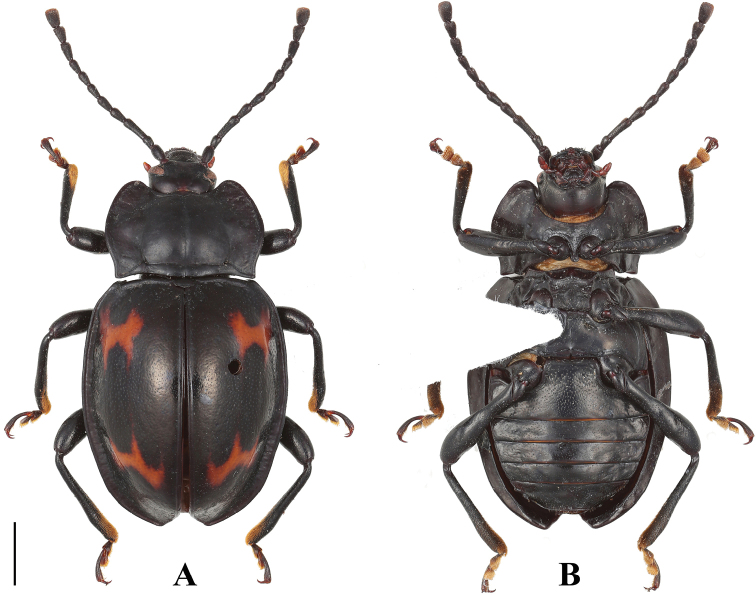
Habitus of *B. denticulatus* sp. nov. (female). **A** dorsal view **B** ventral view. Scale bar: 2 mm.

**Figure 7.**
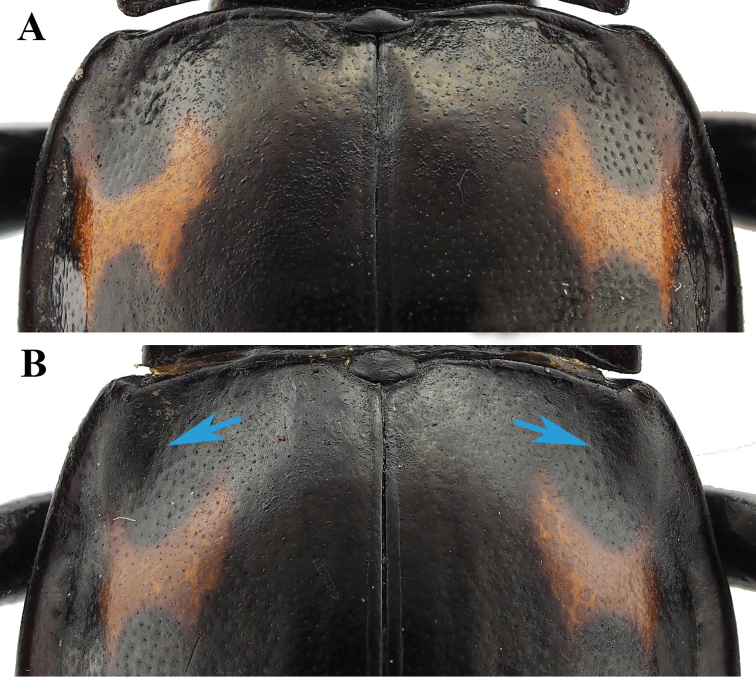
Humeri. **A***B. denticulatus* sp. nov. **B***B. humeralis* sp. nov.

**Figure 8.**
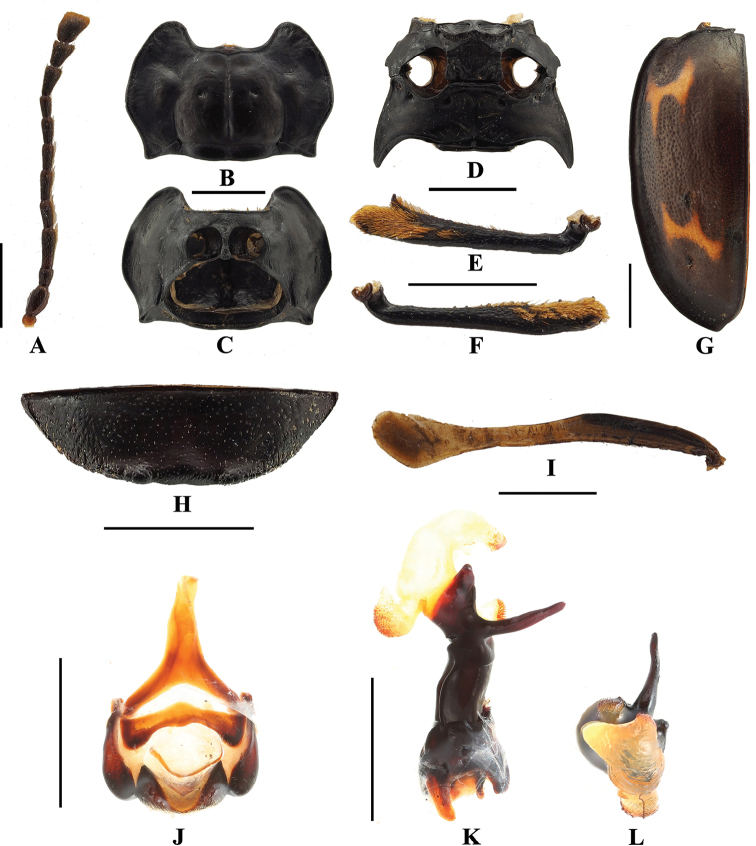
*B. denticulatus* sp. nov. **A** antenna **B** pronotum **C** provenrite **D** meso- and metaventrites **E** male protibia **F** male mesotibia **G** elytron **H** male ventrite V of abdomen **I** hind wing **J** male genital segment **K** aedeagus in lateral view **L** aedeagus in apical view. Scale bars: 1 mm.

**Figure 9.**
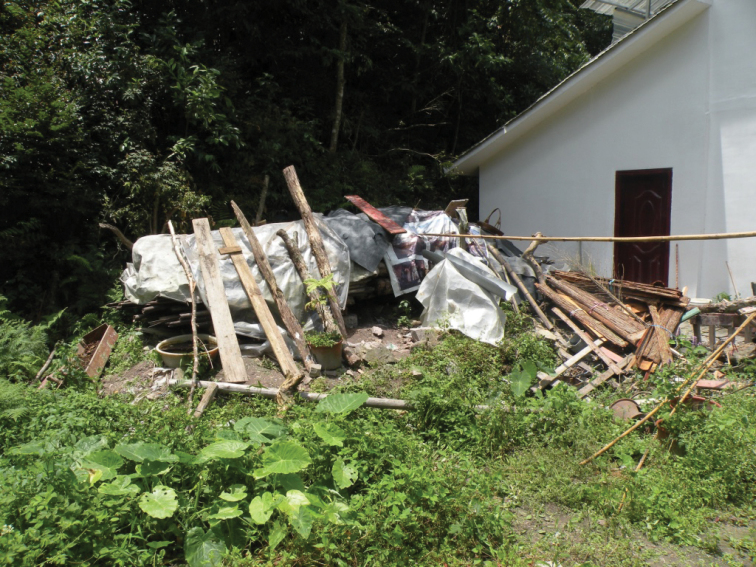
Habitats of *B. denticulatus* sp. nov.

Figs 5 , 6 , 7 , 8 , 9

#### Type material.

Holotype (Fig. 5), male, Guangxi, Jinxiu, Guangxi Dayaoshan Nature Reserve Bureau Yinshan Protection Station, 13.VIII.2015, Ling-Xiao Chang leg. (MHBU). Paratypes (Fig. 6), 1 female, same data as holotype (BJMNH); 1 female, Guangxi, Damingshan, Hao-Yu Liu & Ji-Bin Liang leg. (MHBU); 1 male, Guangxi Prov., Jinxiu County, 16 km, 29.VII.2011, alt. 882–950 m, PENG Zhong leg. (dissected, SHNU); 1 male, Guangxi Prov., Damingshan, Tianping Protect Station, N23.49811, E108.43715, 1230 m, 22.V.2011 N, Xing-Lei Huang Coll. (IZCAS); 1 female, same data except 28.V.2011 (IZCAS); 1 male, Guangxi, Jinxiu, Jiuwanshan, 4.VIII.2015 N, Ling-Xiao Chang leg. (CCLX); 1 male, Guangxi, jinxiu, Yinshan Protection Station, 27.VIII.2016, Yu-Yang Lei leg. (CCLX); 1 male, Guangxi, jinxiu, Dayaoshan, 22-24.IV.2018, Chun-Fu Feng leg. (CCLX); 6 males, 5 females, Guangxi, Damingshan, 1200 m, 28–31.VII.2012, Wen-Xuan Bi leg. (CBWX); 1 male, 1 female, Guangxi, Damingshan, 1200 m, 31.VII.2012, Xiao-Bin Song leg. (CBWX); 1 female, Guangxi, Jinxiu, Yinshanbaohuzhan, 1200 m, 9.VII.2014, Xiao-Bin Song leg. (CBWX); 2 males, 2 females, same data except 10.VII.2014 (CBWX); 1 male, Guangxi, Jinxiu, Laoshanlinchang, 850 m, 18.VII.2014, Xiao-Bin Song leg. (CBWX); 1 male, Guangxi, Nanning, Wuming, Damingshan, N23.49944, E108.44154, 1204 m, 7.VIII.2011, Hai-Tian Song leg. (CSHT).

#### Etymology.

The name refers to the mesotibia serrulated on inner edge in male.

#### Diagnosis.

*Brachytrycherus denticulatus* resembles *B. convexus* in the elytra being strongly convex; posterior elytral maculae transverse, dentate; hind wing reduced to narrow straps. Antenna with club rather narrow (vs. broad in *B. convexus*); *B. denticulatus* pronotum sides strongly curved (vs. weakly rounded and somewhat convergent basally); elytron widest near 1/2 length of elytron (vs. beyond mid-length). *Brachytrycherus denticulatus* is extremely similar to *B. humeralis* sp. nov. in appearance, but the humeri (Fig. 7A) are not distinctly prominent, protibia in male with small sharp tooth near apical 1/4 on inner edge, and mesotibia serrulated on inner edge in male can distinguish *B. denticulatus* from *B. humeralis*.

#### Description.

Length 10.2–13.7 mm, width 5.7–6.9 mm. *Body* broadly oval, approximately 1.8–2.0 times as long as wide; strongly convex; shiny. Colour black with two red-brown maculae on each elytron.

***Head*.** Antenna (Fig. 8A) long and slender, nearly 1/2 body length, with antennomeres 1–8 distinctly longer than wide; scape approximately 4.0 times as long as pedicel; antennomere 3 nearly as long as 4 and 5 combined; antennomere 4 as long as 5, antennomeres 5–8 gradually shorter; club composed of three antennomeres, narrow and moderately flat. Maxilla with terminal palpomere elongate, almost 2.0 times as long as palpomere 3, tapering anteriorly, truncate apically.

***Thorax*.** Pronotum (Fig. 8B) 2.6–3.3 mm long, 4.4–5.4 mm wide; widest behind 1/2 of pronotal length; surface opaque; lateral margins narrowly bordered, sides strongly curved; front angles produced anteriorly, bluntly round; disc weakly convex, with two large round raised areas laterally; transverse wrinkle laterally; median furrow distinct, straight; lateral sulci linear, curved, deep, extending to 1/2 of pronotal length; basal sulcus nearly straight, deep. Prosternal process (Fig. 8C) moderately separates the procoxae, slightly extending beyond coxae; sides curved outwardly, round apically. Mesoventral process (Fig. 8D) transverse, lateral margins barely widening apically, overlapping part of mesocoxae; posterior margin rather straight.

***Elytra*** (Fig. 8G) 7.4–9.3 mm long, 1.3 times as long as wide; 2.8 times as long as and 1.3 times as wide as pronotum, sides curved, widest near 1/2 length of elytron; densely and moderately coarsely punctate; humeri not prominent. Each elytron with two transverse, irregularly shaped red-brown maculae. Anterior elytral macula bowtie-shaped, located behind humerus, its anterior and posterior margins broadly U-shaped and deeply emarginate. Posterior macula crown-shaped, located at apical 1/3, its anterior margin tridentate, posterior margin widely U-shaped and deeply emarginate. Protibia (Fig. 8E) in male with small sharp tooth near apical 1/4 on inner edge, in female without tooth; mesotibia (Fig. 8F) serrulated on inner edge in male, not serrulated in female. Hind wing (Fig. 8I) reduced to narrow straps, oval shape apically, no longer than the elytra.

***Ventrite* V** (Fig. 8H) with lateral margins gently converging posteriorly; posterior margin in male with some small lateral tubercles, one raised area medially; in female ventrite V with posterior margin simple, weakly emarginate medially. Male genital segment (Fig. 8J) with paired apophyses fused along nearly 1/3 of its length basally; dorsal plate undivided; additional, internal, V-shaped sclerite present.

***Aedeagus*** (Fig. 8K, L) rather long, heavily sclerotized, straight. Median lobe branched apically; branch moderately long and rather straight, flat and round apically. Tegmen placed basally, comparatively large, ring-shaped; parameres rather large, rectangle, fused with tegmen.

#### Biology and ecology.

The adults were collected by hand collected from a large pile of dead bamboos in the day (Fig. 9).

### Brachytrycherus humeralis

Taxon classificationAnimaliaColeopteraEndomychidae

Chang & Bi sp. nov.

740E22C8-AC2A-5998-88C2-CADF62BF5AD1

http://zoobank.org/99D5DA5E-DB6E-4FB1-A254-FEFBF472B40A

**Figure 10.**
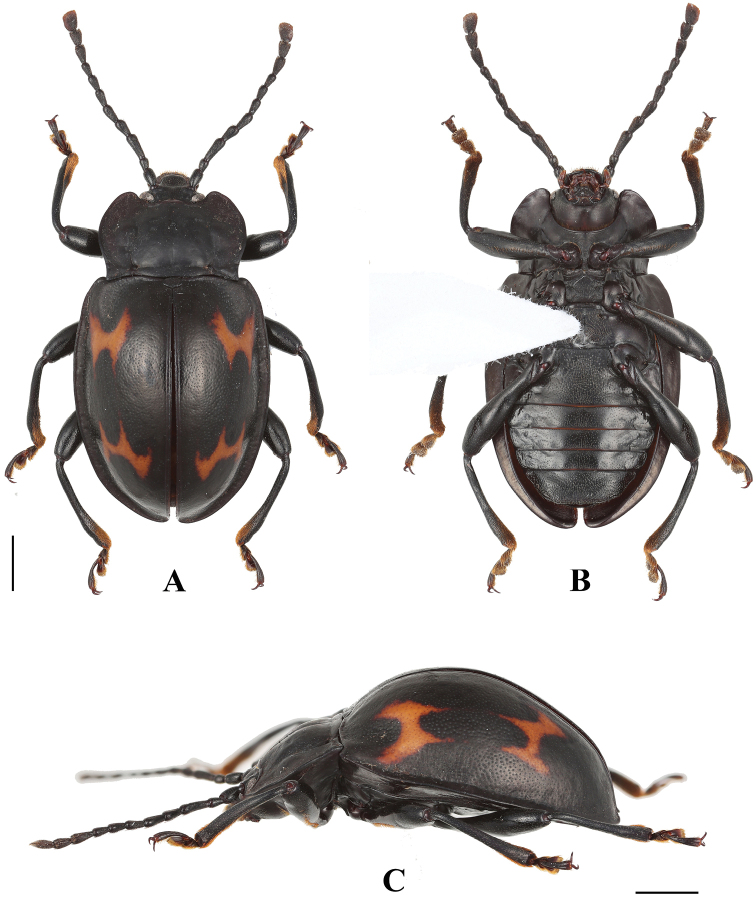
Habitus of *B. humeralis* sp. nov. (male). **A** dorsal view **B** ventral view **C** lateral view. Scale bars: 2 mm.

**Figure 11.**
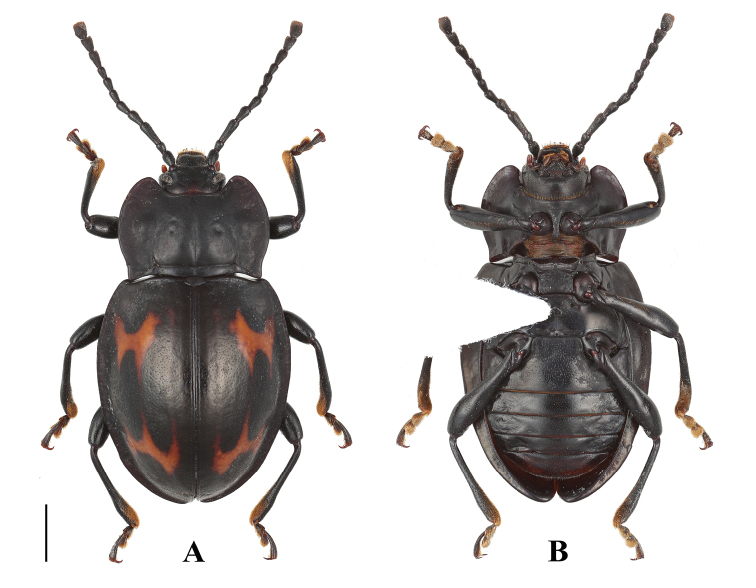
Habitus of *B. humeralis* sp. nov. (female). **A** dorsal view **B** ventral view. Scale bar: 2 mm.

**Figure 12.**
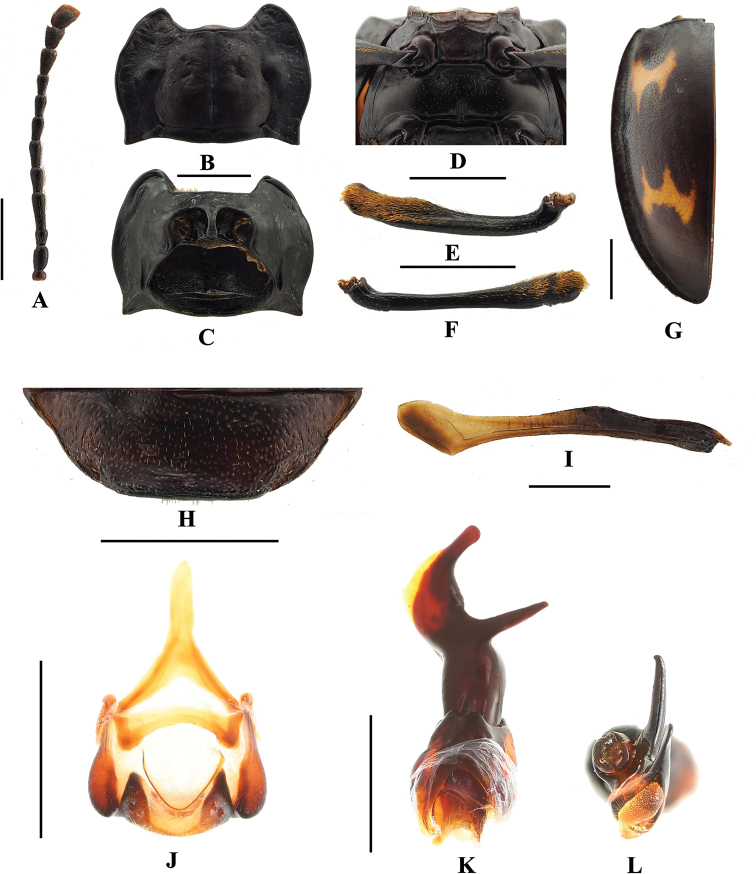
*B. humeralis* sp. nov. **A** antenna **B** pronotum **C** proventrite **D** meso- and metaventrites **E** male protibia **F** male mesotibia **G** elytron **H** male ventrite V of abdomen **I** hind wing **J** male genital segment **K** aedeagus in lateral view **L** aedeagus in apical view. Scale bars: 1 mm.

**Figure 13.**
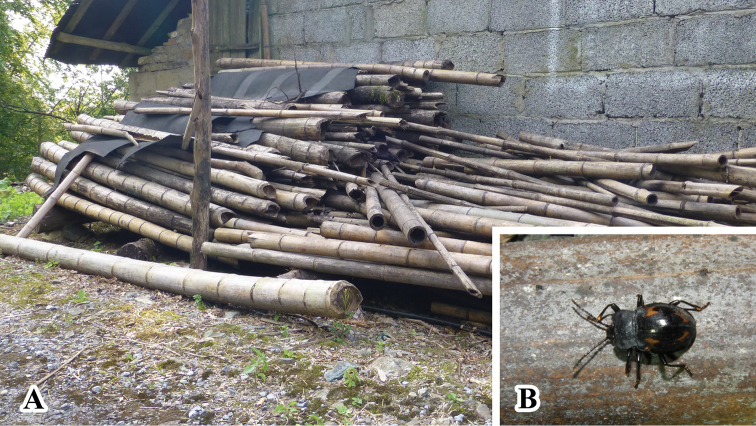
Habitats of *B. humeralis* sp. nov. **A** large pile of dead bamboos in Guangxi, China **B** adult of *B. humeralis* sp. nov.

Figs 10 , 11 , 12 , 13

#### Type material.

Holotype (Fig. 10), male, Guangxi, Huanjiang, Yangmeiao Protection Station, 4.VIII.2015 N, Ling-Xiao Chang leg. (MHBU). Paratypes (Fig. 11), 1 female, same data as holotype (BJMNH); 1 male, same data as holotype (CCLX); 1 male, Guangxi, Damingshan, Longteng, Power Station, N23.49811, E108.43715, 1230 m, 20.V.2011 N, Xing-Lei Hhang leg. (IZCAS); 1 male, same data except dissected (IZCAS); 1 female, Guangxi, Jinxiu, Shengtangshan, 700 m, 19.V.1999, Fu-Sheng Huang leg. (I0Z(E)1172359, IZCAS). 1 male, Guangxi, Jinxiu, Yinshan, 27.VIII.2016, Yu-Yang Lei leg. (CCLX).

#### Etymology.

The name refers to the humeri with a distinct raised oval area.

#### Diagnosis.

*Brachytrycherus humeralis* resembles *B. convexus* in the elytra being strongly convex; posterior elytral maculae transverse, dentate; hind wing reduced to narrow straps. However, they can be differentiated by *B. humeralis* with the antennal club that is rather narrow (vs. broad); the pronotum sides are strongly curved (vs. weakly rounded and somewhat convergent basally); and the elytra are widest near 1/2 length of elytron (vs. beyond mid-length). In addition, *B. humeralis* is extremely similar to *B. denticulatus* sp. nov. in appearance. The humeri (Fig. 7B) are distinctly prominent, the protibia in males is without a tooth, raised near apical 1/3 on inner edge, and the simple mesotibia in males can distinguish *B. humeralis* from *B. denticulatus*.

#### Description.

Length 12.5–12.7 mm, width 6.6–7.0 mm. *Body* broadly oval, approximately 1.8–1.9 times as long as wide; strongly convex; shiny. Colour black with two red-brown maculae on each elytron.

***Head*.** Antenna (Fig. 12A) long and slender, nearly 1/2 body length, with antennomeres 1–8 distinctly longer than wide; scape approximately 3.0 times as long as pedicel; antennomere 3 nearly as long as 4 and 5 combined; antennomere 4 as long as 5, antennomeres 5–8 gradually shorter; club composed of three antennomeres, narrow and moderately flat. Maxilla with terminal palpomere elongate, almost 1.5 times as long as palpomere 3, tapering anteriorly, truncate apically.

***Thorax*.** Pronotum (Fig. 12B) 2.9–3.2 mm long, 5.1–5.3 mm wide; widest 1/2 of pronotal length; surface opaque; lateral margins narrowly bordered, sides strongly curved; front angles produced anteriorly, bluntly round; disc weakly convex, with two large round raised areas laterally; transverse wrinkle laterally; median furrow distinct, straight; lateral sulci linear, curved, deep, extending to 1/2 of pronotal length; basal sulcus nearly straight, deep. Prosternal process (Fig. 12C) moderately separates the procoxae, slightly extending beyond coxae; sides curved outwardly, round apically. Mesoventral process (Fig. 12D) transverse, lateral margins weakly widening apically and overlapping part of mesocoxae, in some specimens hardly widening apically; posterior margin rather straight.

***Elytra*** (Fig. 12G) 8.9–9.6 mm long, 1.3 times as long as wide; 2.8 times as long as and 1.3 times as wide as pronotum, sides curved, widest near 1/2 length of elytron; densely and moderately coarsely punctate; humeri distinctly prominent. Each elytron with two transverse, irregular red-brown maculae. Anterior elytral macula bowtie-shape, located behind humeri, its anterior and posterior margin widely U-shaped and deeply emarginate. Posterior macula crown-shaped, located at apical 1/3, its anterior margin tridentate, posterior margin widely U-shaped and deeply emarginate. Protibia (Fig. 12E) in male raised near apical 1/3 on inner edge, in female not raised; mesotibia (Fig. 12F) simple. Hind wing (Fig. 12I) reduced to narrow straps, oval shape apically, no longer than the elytra.

***Ventrite* V** (Fig. 12H) with lateral margins gently converging posteriorly; posterior margin in male widely raised medially; in female ventrite V with posterior margin simple, weakly emarginate medially. Male genital segment (Fig. 12J) with paired apophyses fused along nearly 1/3 of its length basally; dorsal plate undivided; additional, internal, V-shaped sclerite present.

***Aedeagus*** (Fig. 12K, L) rather long, heavily sclerotized, distinctly curved outwardly near 1/2 of length, and with one branch, rather short and straight, weakly acute apically. Median lobe branched apically, short and straight, flat and widely round apically. Tegmen placed basally, comparatively large, ring-shaped; parameres rather large, fused with tegmen.

#### Biology and ecology.

The adults were collected by hand collected from a large pile of dead bamboos at night (Fig. 13).

### Brachytrycherus conaensis

Taxon classificationAnimaliaColeopteraEndomychidae

Chang, Bi & Ren, 2016

FFC9182B-DE4E-5E99-83B6-996E63D8BF42

**Figure 14.**
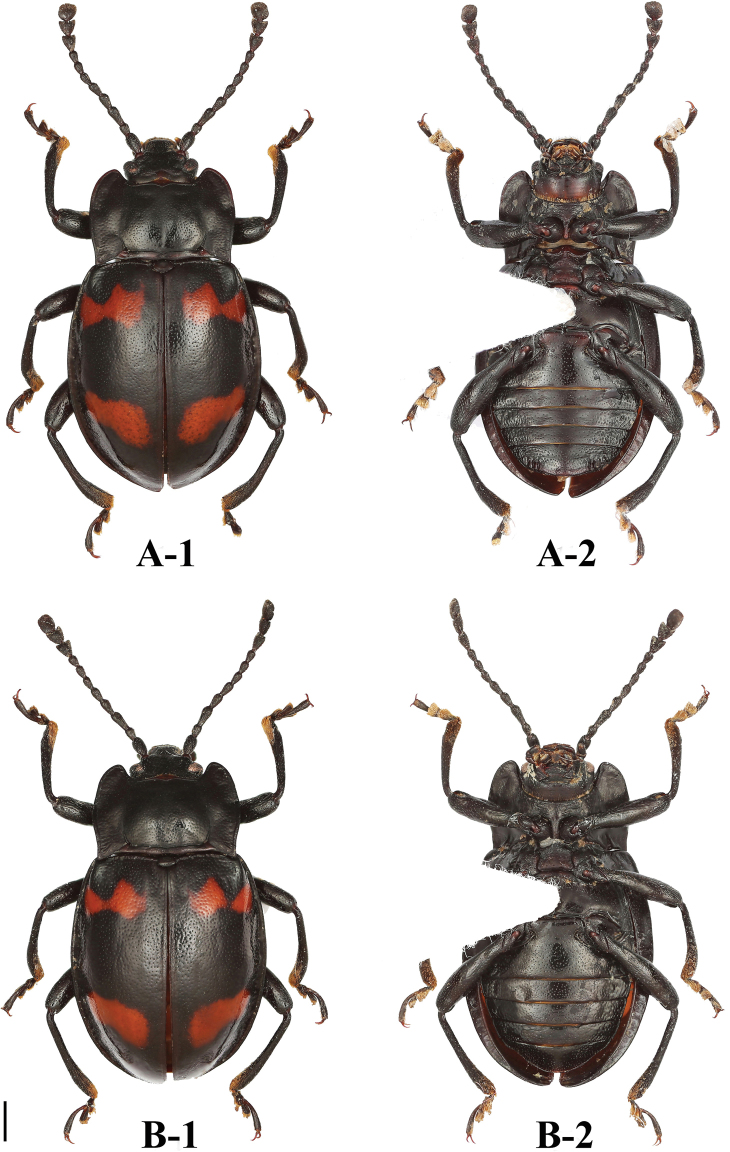
Type specimens of *B. conaensis*. **A** male holotype **B** female paratype **1** dorsal view **2** ventral view. Scale bar: 1 mm.

Figs 14 , 16D, E , 20 , 21 , 22 , 23 , 24A

Brachytrycherus conaensis Chang et al., 2016: 139.

#### Diagnosis.

*Brachytrycherus conaensis* is similar to *B. madurensis* in appearance but can be differentiated by each elytron bearing three maculae, anterior two maculae nearly rhomboid in shape, sometimes connected to each other, and the anterior and posterior elytral maculae without dentition.

#### Length.

8.2–8.3 mm; width: 4.5–4.7 mm.

#### Type material.

Holotype, male, Xizang, Cona, Lexiang, 2500–2600 m, 20-30.VI.2013, Wen-Xuan Bi leg. (MHBU). Paratypes, 1 female, same data as holotype; 2 females, Xizang, Medog, Beibeng, Gelincun, 1700 m, 3.VIII.2014, Wen-Xuan Bi leg. (CBWX); 3 males, 7 females, Xizang, Cuona, Lexiang, 2500 m, 6.VIII.2010, Wen-Xuan Bi leg. (CBWX); 5 males, 6 females, same data except 15.VII.2011 (CBWX); 26 males, 11 females, same data except 29–30.VI.2013 (CBWX); 1 male, 1 female, same data except (MZPW); 18 males, 1 female, same data except 2500–2600 m, 20-30.VI.2013 (CBWX); 1 female, same data except 2700 m, 18.VI.2013 (CBWX).

#### Type locality.

China (Xizang).

#### Distribution.

China (Xizang).

#### Biology and ecology.

Almost all individuals were found active on fence, woodpile or timber piles within the village and its surrounding area at night (Fig. 16D, E)). Some larvae and adults were found (sometimes at the same time) feeding on the surface of the perithecia or spores of *Daldinia concentrica* (Xylariaceae) (Fig. 16D), seeming to prefer the asexual phase; however, individuals were also found on mature ascocarps (Chang et al. 2016).

### Brachytrycherus curviantennae

Taxon classificationAnimaliaColeopteraEndomychidae

Chang, Bi & Ren, 2016

A6F55371-534B-5DB0-9D3B-1CF257D8D501

Figs 15 , 16A, C

Brachytrycherus curviantennae Chang et al., 2016: 139.

#### Diagnosis.

*Brachytrycherus curviantennae* is similar to *B. humeralis* and *B. denticulatus* sp. nov. in both bodies being broadly oval, elytral maculae transverse, pronotum sides strongly curved. However, antennomere 3 distinctly curved outwards and elytral maculae nearly cymbiform can distinguish *B. curviantennae* from all its congeners.

#### Length.

8.5–9.4 mm; width: 5.1–5.2 mm.

#### Type material.

Holotype, male, Xizang, Medog, 1500 m, 20.VIII.2013, Wen-Xuan Bi leg. (SHEM). Paratypes, 1 female, Xizang, Medgo, Beibeng, Gelincun, 3.VIII.2014, Wen-Xuan Bi leg. (MHBU); 1 female, Xizang, Medgo, Beibeng, Gelincun, 3.VIII.2014, Wen-Xuan Bi leg. (CBWX).

#### Type locality.

China (Xizang).

#### Distribution.

China (Xizang).

#### Biology and ecology.

The male was hand collected by simple searching, as it is active on branches at night (Fig. 16C). Two females were collected by shaking the tree from a large clump of dead wood of Fagaceae plants (Fig. 16A) (Chang et al. 2016).

**Figure 15.**
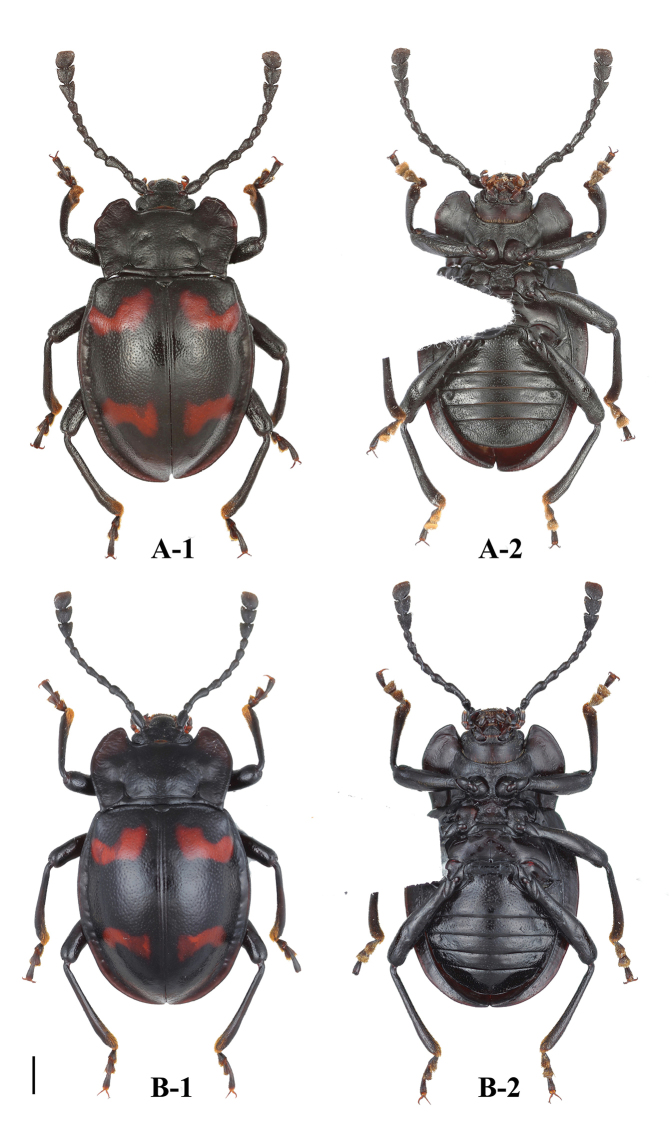
Type specimens of *B. curviantennae*. **A** male holotype **B** female paratype; **1** dorsal view; **2** ventral view. Scale bar: 1 mm.

**Figure 16.**
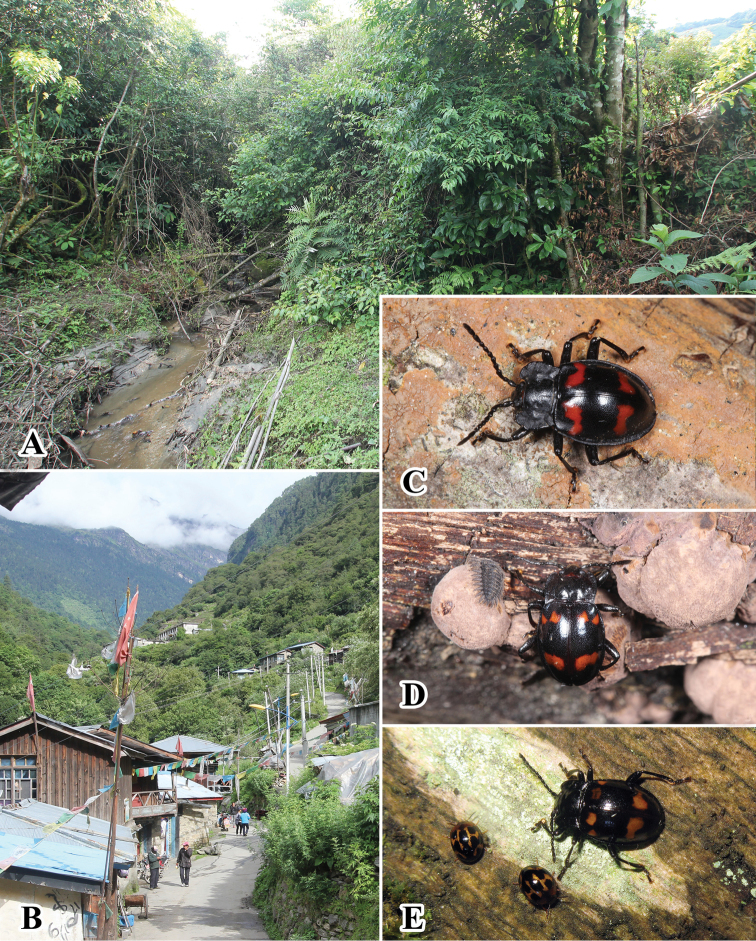
Habitats of *B. conaensis* and *B. curviantennae*. **A** large clump of Fagaceae plants of collecting site in Xizang, China **B** village of collecting site in Xizang, China **C** male of *B. curviantennae* (arranged) **D** male of *B. conaensis* and larva on the wood pile **E** female of *B. conaensis* feeding on the lichen growing on wood.

### Brachytrycherus femoralis

Taxon classificationAnimaliaColeopteraEndomychidae

(Arrow, 1928)

5CB04B3B-AA4E-51AE-9469-FB7028FF6A60

Figs 17 , 18 , 19

Engonius femoralis Arrow, 1928: 347.

#### Diagnosis.

*Brachytrycherus femoralis* can be separated from all its congeners by having three elytral maculae; sides of elytra strongly converging from its 1/2 length towards apex, lateral margins moderately widely flattened, not vanishing at apex.

#### Material examined.

**China: Guangxi Province**: Jinxiu, Yinshan Protection Station, 27.VI.2016, Yu-Yang Lei leg. (1 male, 2 females, CCLX); Huanjiang Yangmeiao Protection Station, 15.VIII.2016, Ling-Xiao Chang leg. (2 males, CCLX); Jinxiu, Dayaoshan, 22–24.IV.2018, Chun-Fu Feng leg. (2 females, CCLX); Jinxiu, Yinshan Protection Station, 1500 m, 12.VIII.2015, Ling-Xiao Chang leg. (2 males, 3 females, MHBU); Jinxiu, Dayaoshan, 17.V.2014, Zhi-Lin Chen leg. (1 female, MHBU); Longsheng, Huaping, 15.X.2005, Ji-Liang Wang & Chao Gao leg. (1 female, MHBU).

#### Description.

Length 9.4–11.2 mm, width 4.3–5.4 mm. *Body* oval, about 2.1–2.3 times as long as wide; moderately convex; shiny. Colour black with purple sheen, three orange-red maculae on each elytron.

***Head*.** Antenna long and rather slender, nearly 1/2 body length, with antennomeres 1–8 distinctly longer than wide; scape approximately 4.0 times as long as pedicel; antennomere 3 as long as 4 and 5 combined; antennomeres 4 nearly as long as 5, antennomeres 5–8 gradually shorter; club composed of three antennomeres, moderately broad, flat, loose. Maxilla with terminal palpomere longer than wide, slightly longer than palpomere 3, tapering anteriorly, truncate apically.

***Thorax*.** Pronotum 2.1–2.2 mm long, 3.5–4.2 mm wide; widest near 1/2 of pronotal length; finely and densely punctate; lateral margins rather narrowly bordered, sides undulate; front angles produced anteriorly, rather acute; disc weakly convex, two small round raised area laterally; transverse wrinkle and inflexed laterally; median furrow shallow, extending to 1/2 length of pronotum; lateral sulci linear, deep, extending to basal 1/3 length of pronotum; basal sulcus weakly undulate, deep. Prosternal process moderately separates procoxae; not extending beyond coxae; sides nearly parallel, expanded apically; posterior margin in male truncate and emarginate in female. Mesoventral process transverse, lateral margins weakly widening apically and overlapping part of mesocoxae; posterior margin nearly straight.

***Elytra*** 6.3–7.3 mm long, 3.0–3.3 times as long as pronotum and 1.2–1.3 times as wide as pronotum, sides curved, widest near 1/2 length of elytron; finely and densely punctate; humeri prominent. Each elytron with three irregular orange-red maculae. Anterior two elytral maculae located near basal 1/4, lateral maculae oval, almost confined to umbo; medial macula nearly round, larger than lateral one, sometimes narrowly connected. Posterior macula located near apical 1/4, weakly transverse, nearly cloud-form, outer sides far from elytral lateral margin, inner margin of macula far from elytral suture. Protibia in male with rather long sharp tooth near 1/2 length on inner edge, in female without tooth; mesotibia in male with small sharp tooth behind 1/2 length on inner edge, and then abruptly curved to apex, in female without tooth.

***Ventrite* V** with lateral margins gently converging posteriorly; posterior margin truncate in male and weakly curved in male medially.

***Aedeagus*** (Fig. 5) rather long, heavily sclerotized, straight. Median lobe one branched apically; branch long and strongly reflexed upwardly, acute apically. Tegmen basal, comparatively large, ring-shaped.

#### Distribution.

China (Guangxi), Laos, Vietnam (Tonkin). First records from China.

#### Type locality.

Lectotype: Laos, 1 male; Syntype: Vietnam (Tonkin), 1 male.

#### Biology and ecology.

The adults were found active and feeding on the mould growing on dead bamboos at night (Fig. 18). The adults and larvae were brought back and placed in artificial conditions to rear. The last instar larvae pupated on surface of dead bamboos, from their pupal stage to matured to adults in approximately seven to nine days (Fig. 19).

**Figure 17.**
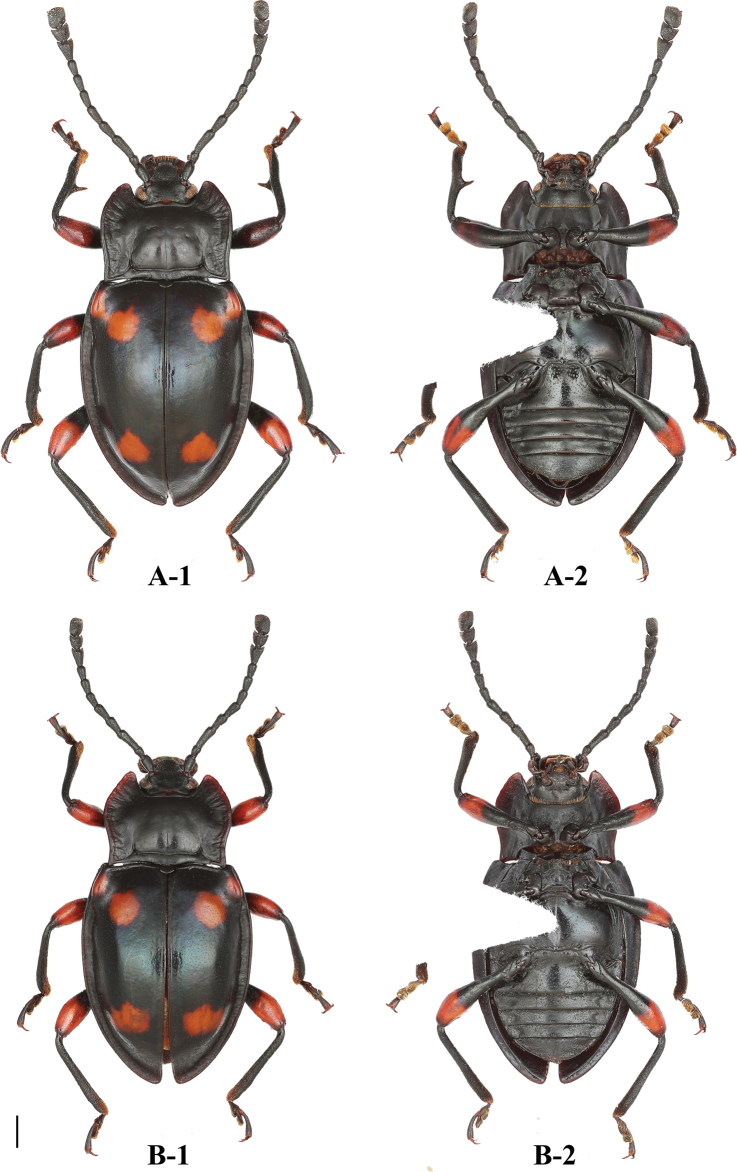
Habitus of *B. femoralis***A** male **B** female; **1** dorsal view **2** ventral view. Scale bar: 1 mm.

**Figure 18.**
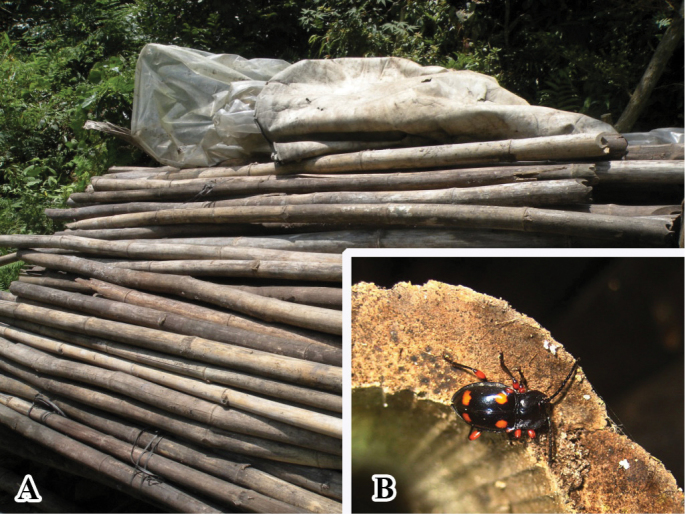
Habitats of *B. femoralis*. **A** large pile of dead bamboos in Guangxi, China **B** adult of *B. femoralis* sp. nov. feeding on the mold growing on dead bamboos.

**Figure 19.**
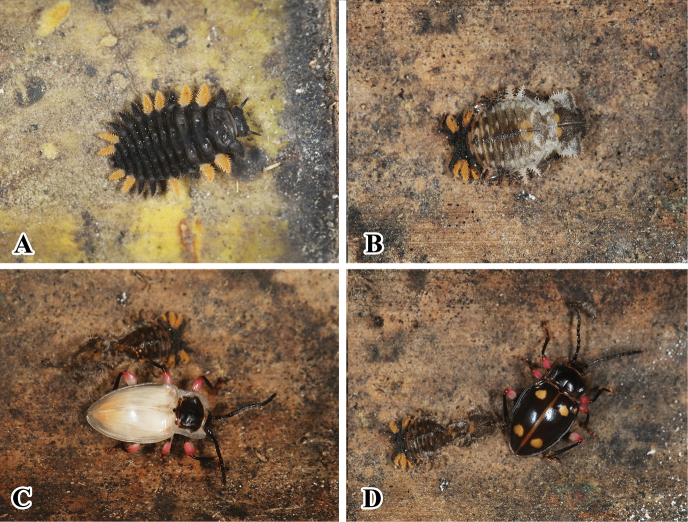
Living adults, larva, and pupae of *B. femoralis* in artificial conditions. **A** last instar larvae **B** pupae **C, D** newly emerged adult.

### Key to the species of *Brachytrycherus* known in mainland China (adapted from Chang et al. 2016)

**Table:** 

1	Antennomere 3 distinctly outwardly curved	***B. curviantennae***
–	Antennomere 3 straight	**2**
2	Elytron strongly convex, hind wing reduced to narrow straps	**3**
–	Elytron moderately convex, hind wing fully developed	**4**
3	Elytral humerus distinctly prominent, protibia in male without sharp tooth	***B. humeralis* sp. nov.**
–	Elytral humerus not prominent, protibia in male with sharp tooth	***B. denticulatus* sp. nov.**
4	Elytral sides strongly converging from its 1/2 length towards apex, lateral margins moderately widely flattened, not vanishing at apex	***B. femoralis***
–	Elytral sides gradually converging from its 1/3 length towards apex, lateral margins rather narrowly bordered, vanishing at apex	**5**
5	Elytron with anterior two maculae nearly rhomboid, sometimes connected to each other	***B. conaensis***
–	Elytron with anterior two maculae nearly oval or round, rather remote from each other	***B. bipunctatus* sp. nov.**

**Figure 20.**
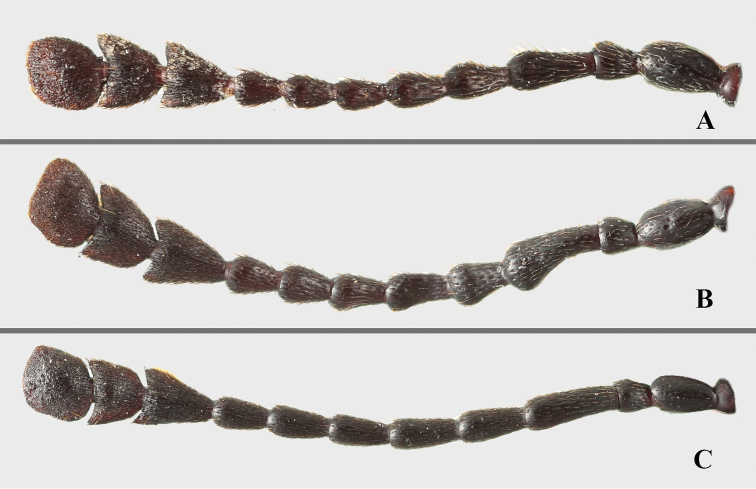
Left antenna of *Brachytrycherus*. **A***B. conaensis***B***B. curviantennae***C***B. femoralis*.

**Figure 21.**
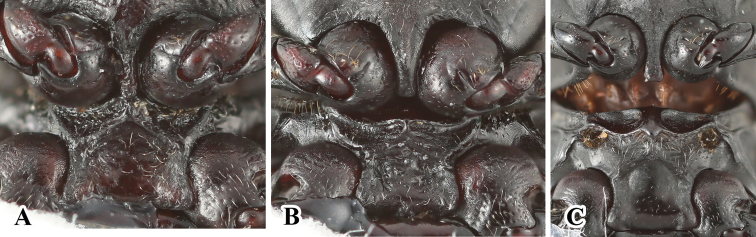
Prosternal and intercoxal processes of males. **A***B. conaensis***B***B. curviantennae***C***B. femoralis*.

**Figure 22.**
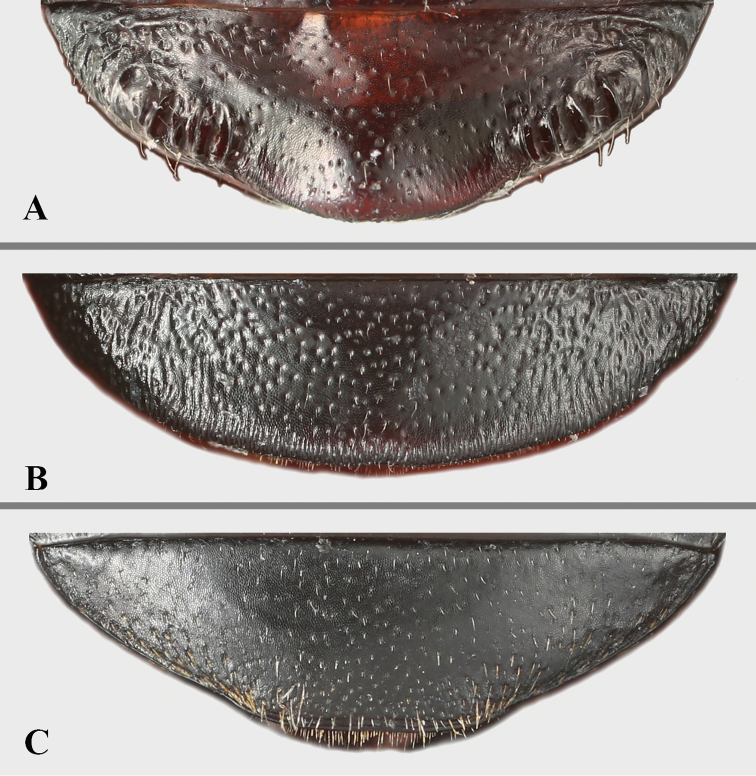
Abdomen with ventrite V of males. **A***B. conaensis***B***B. curviantennae***C***B. femoralis*.

**Figure 23.**
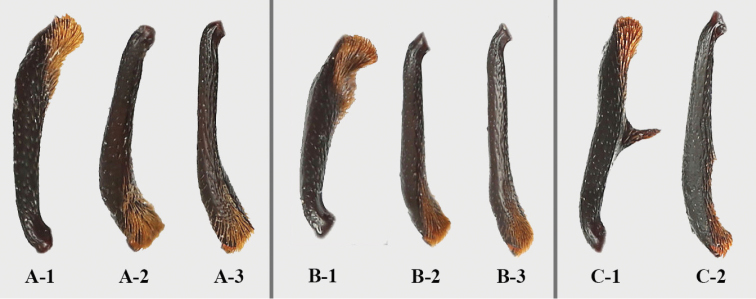
Left tibia of *Brachytrycherus* (male). **A***B. conaensis***B***B. curviantennae***C***B. femoralis*; 1 protibia; 2 mesotibia; 3 metatibia.

**Figure 24.**
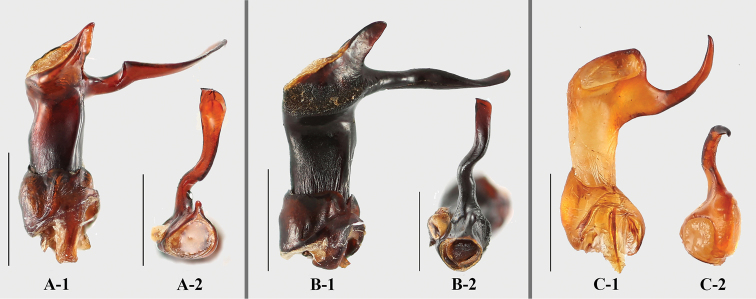
Aedeagus of *Brachytrycherus*. **A***B. conaensis***B***B. curviantennae***C***B. femoralis*; **1** lateral view; **2** apical view. Scale bars: 1 mm.

## Discussion

The genus *Brachytrycherus* belongs to the Amphisternus group of Lycoperdininae; the monophyly of this group was defined by Tomaszewska (2005), based on shared characteristics. However, in some specimens (both new species and *B. femoralis*) the mesoventrite intercoxal process sides are weakly widened apically. The shape of the mesoventrite intercoxal process is not stable; thus, it may not be appropriate as a character used to defined the Amphisternus group.

The unique character for *Brachytrycherus* is a sexual dimorphism in the shape of the prosternal process in some species: for example, the prosternal process in male *B. bipunctatus* sp. nov. is very narrow, the sides are weakly curved outwardly, rounded apically; females are wider than males, the sides are nearly straight, and weakly truncate apically. This character is observed for the first time in the Endomychidae.

## Supplementary Material

XML Treatment for Brachytrycherus

XML Treatment for Brachytrycherus bipunctatus

XML Treatment for Brachytrycherus denticulatus

XML Treatment for Brachytrycherus humeralis

XML Treatment for Brachytrycherus conaensis

XML Treatment for Brachytrycherus curviantennae

XML Treatment for Brachytrycherus femoralis
